# RoLaCaRT-1: pilot randomised phase II study of robotic vs laparoscopic hemicolectomy for right colon cancer

**DOI:** 10.1007/s00464-025-12400-1

**Published:** 2025-12-10

**Authors:** Andrew R. L. Stevenson, Jim S. Khan, Kate Wilson, Rachel L. O’Connell, Stephen Pillinger, Craig Lynch, Nader K. Francis, Nathan J. Curtis, Danilo Miskovic, Andrew D. Clouston, Gregory C. Miller, Carina F. K. Chow, Satish Warrier, Alexander G. Heriot, Darren Tonkin, Stephen Bell, Mark D. Muhlmann, Kirk K. S. Austin, Christopher J. Gillespie, David W. Larson, Larissa Temple, Alessio Pigazzi, Rebecca Mercieca-Bebber, John Simes

**Affiliations:** 1 Royal Brisbane and Women’s Hospital, Brisbane, QLD Australia; 2 Faculty of Health, Medicine and Behavioural Sciences, University of Queensland, Brisbane, QLD Australia; 3 Faculty of Medicine and Health, National Health and Medical Research Council Clinical, Trials Centre, University of Sydney, Camperdown, NSW Australia; 4 St Vincent’s Private Hospital Northside, Level 1, 627 Rode Road, Chermside, Brisbane, QLD 4032 Australia; 5 Department of Colorectal Surgery, Portsmouth Hospitals University NHS Trust, Portsmouth, UK; 6 Faculty of Science and Health, University of Portsmouth, Portsmouth, UK; 7 Department of Robotic Colorectal Surgery, Sydney Adventist Hospital, Sydney, NSW Australia; 8 ANU School of Medicine and Psychology, The Australian National University, Canberra, ACT Australia; 9 The Griffin Institute, Northwick Park, UK; 10 St Marks Hospital, London, UK; 11 Department of Surgery, Dorset County Hospital NHS Foundation Trust, Dorchester, UK; 12 Envoi Specialist Pathologists, Brisbane, QLD Australia; 13 Division of Cancer Surgery, Peter MacCallum Cancer Centre, Melbourne, VIC Australia; 14 The Sir Peter MacCallum Department of Oncology, University of Melbourne, Melbourne, VIC Australia; 15 Colorectal Department, The Queen Elizabeth Hospital, Adelaide, SA Australia; 16 Cabrini Malvern, Melbourne, VIC Australia; 17 Department of Surgery, Monash University, Melbourne, VIC Australia; 18 Colorectal Surgical Department, Prince of Wales Hospital, Sydney, NSW Australia; 19 Department of Colorectal Surgery Royal Prince Alfred Hospital, Sydney, NSW Australia; 20 Surgical Outcomes Research Centre (SOuRCe), Sydney, NSW Australia; 21 Sydney Local Health District, The Institute of Academic Surgery at RPA, Sydney, NSW Australia; 22 Department of Surgery, Mater Hospital, Brisbane, QLD Australia; 23 Division of Colorectal Surgery, Mayo Clinic, Rochester, MN USA; 24 Colorectal Surgery, University of Rochester Medical Centre, New York, NY USA; 25 Weill Cornell Medical Centre, New York, NY USA

**Keywords:** Colon cancer, Robotic surgery, Laparoscopic surgery, Right hemicolectomy, Randomised controlled trial, Comprehensive complication index

## Abstract

**Background:**

Previous stage I-III colon cancer trials demonstrated improved peri-operative and similar long-term oncological outcomes with minimally invasive approaches compared with open surgery. Subsequent technical developments, including intracorporeal anastomosis, complete mesocolic excision (CME), refined pathologic grading systems and robotic approaches are increasingly used despite minimal supporting evidence. This international phase II trial aims to assess patient, surgical and pathological outcomes following robotic (RRHC) with laparoscopic right hemicolectomy (LRHC) for right sided colon cancer.

**Methods:**

Prospective, international multicentre pilot randomised phase II trial for patients with right colon adenocarcinoma, randomised 2:1 to robotic (RRHC) or laparoscopic (LRHC) surgery performed by accredited surgeons. Primary outcome was 90-day surgical morbidity measured using the Comprehensive Complication Index (CCI). Standardised pathology reviews, patient reported quality of life and surgeon task load index data was captured.

**Results:**

RRHC vs. LRHC surgery allocation was 19 vs. 10, mean operative time 3.8 vs. 3.1 h with no operative mortality, all had R0 resection, CME rate 53 vs. 50% and intact mesocolon 94 v 100%. CCI was significantly lower at 30 and 90 days after RRHC (median (Q1, Q3): 0 (0, 9) vs. 21 (21, 26); median difference: 21; 95% CI 9–26; *p* = 0.005 at 30 days; *p* = 0.008 at 90 days). No significant differences in pathological nor quality of life data was observed. Surgeons task load data tended to favour robotic procedures. Median follow-up was 6 months. Limited funding led to early study closure.

**Conclusions:**

RRHC improved short-term surgical morbidity and surgeon physical demand and performance whilst obtaining similar quality of life and pathological specimen quality compared to LRHC for right sided colon cancer, supporting the case for larger randomised trials.

**Trial registration:**

ACTRN12620001378910

**Graphical Abstract:**

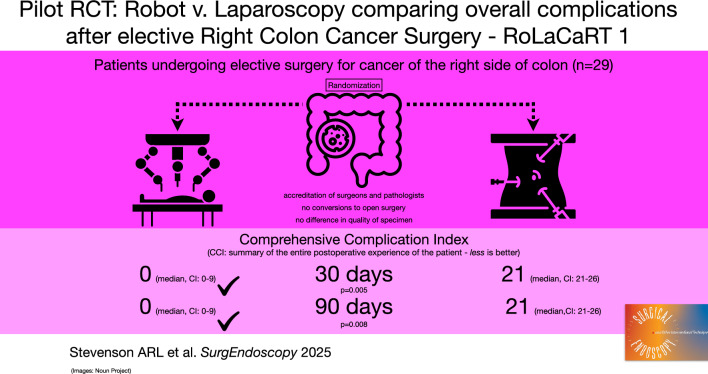

**Supplementary Information:**

The online version contains supplementary material available at 10.1007/s00464-025-12400-1.

Cancer of the colon is the third commonest type worldwide [1]. Right colon cancers have a lower cancer-specific and overall survival compared with left colon cancers despite similar nodal harvest and rate of systemic therapy [2, 3]. Surgery remains the main curative treatment for stage I-III colon cancer with minimally invasive approaches recommended over laparotomy as first choice due to improved peri-operative outcomes with similar long-term oncological outcomes [4–8].

Over the past decades, a number of technical refinements have been proposed including intracorporeal anastomosis (ICA), complete mesocolic excision (CME), updated pathologic grading systems and the use of robotic platforms. There remains varying rates of adoption of these techniques for right hemicolectomy, partly due to the lack of supporting evidence, but possibly also due to their increased complexity.

Robotic platforms were specifically designed to overcome the limitations of laparoscopy for surgeries in limited spaces such as the pelvis and where straight instruments may present ergonomic difficulties. Robotics may permit finer tissue dissection and facilitate wider uptake of ICA and CME potentially improving the outcomes of minimally invasive right hemicolectomies [9–19]. Despite the rapid uptake of robotics in colorectal surgery, RRHC has not been yet robustly evaluated [20]. The available studies comparing robotic right hemicolectomy (RRHC) with laparoscopy (LRHC) reported moderate morbidity, length of stay, and conversion rate benefits with an increased operative times and costs, longer learning curves, and similar clinical outcomes [21–24].

CME is an embryologically based technique which has gained interest in the treatment of colon cancers following the seminal report from Hohenberger where 5-year local recurrences rates decreased (6.5% to 3.6%) with improved cancer-related 5-year survival rates (82.1% to 89.1%) [25]. CME is technically demanding and so usually performed with an open approach. It is possible through improved visualization and greater surgical dexterity that robotics could assist safe minimally invasive CME delivery [26–29]. To our knowledge, no trials on RRHC with CME have been yet conducted. Therefore, RoLaCaRT-1 was designed as an exploratory Phase II RoLaCaRT-feasibility randomised trial to inform the design and assess the practicality of delivering a future definitive trial to test the underlying hypothesis that robotic assisted resection for cancer of the right colon improves outcomes compared to laparoscopy.

## Materials and methods

### Study design

RoLaCaRT-1 was a prospective, multinational randomised, phase II trial designed in collaboration with centres in Australia, the UK and the US. It was performed in Australia and the UK between August 2021 and December 2022. Participants were adults that had provided written informed consent, with ECOG performance status 0–2, ASA I-III and > 1 year life expectancy, undergoing elective surgery with curative intent for endoscopically and biopsy confirmed adenocarcinoma of the caecum, ascending or proximal transverse colon. Patients were excluded if they had cT4 disease, received neoadjuvant chemotherapy, were undergoing emergency, unplanned, delayed (> 40 days from consent), palliative intent, non-resectional and/or no anastomosis surgery or if the pre-operative plan was to perform an extended right hemicolectomy or any other more extensive colonic or synchronous procedure. Upon recruitment participants underwent central, electronic 2:1 randomization to robotic or laparoscopic right hemicolectomy stratified by sex, BMI (≤ 30 v > 30), tumour site (caecum/ascending colon vs. hepatic flexure/proximal transverse colon) and surgeon. Patients and outcome assessors remained blinded to allocation arm. The trial was prospectively registered (ACTRN12620001378910) with central ethical approval obtained from the Sydney Local Health District Ethics Review Committee (ref: X19-0463). Local ethical approvals were obtained in each study centre and country before commencement.

### Quality assurance measures

#### Surgeon accreditation

High volume colorectal units with established robotic surgery programs were identified and invited to participate in the trial. Participating surgeons must have performed at least 30 independent laparoscopic and at least 30 independent robotic operations for colorectal cancer. Unedited anonymised videos of a robotic and a laparoscopic right hemicolectomy, performed within the last 6 months were submitted with matched pathological data which was scored using the Benz classification [30]. A Surgeon Accreditation Committee (SAC) comprised of six surgeons (SP, CL, AS, DM, NF, NC) from the Trial Management Committee (TMC) performed structured, standardised blinded assessments using the validated Laparoscopic Competency Assessment Tool [31] (LCAT). As CME is not standard practice and the definition somewhat variable, the SAC reached consensus that an acceptable dissection and vascular pedicle control in this study was defined by positive identification of the ileocolic/superior mesenteric vein junction and visualization of the head of the pancreas. The minimum composite LCAT score for accreditation was 2.7 with a Benz classification of 0 or 1a. The type of anastomosis performed in the study was surgeon preference. However, to perform an ICA within the trial, surgeon credentialing required experience of a minimum of 5 independent ICA including both laparoscopic and robotic systems, with at least one ICA present on the pre-trial submitted case videos. Surgical workshops were planned however due to the impact of Covid-19 a series of remote training sessions were held via teleconference instead.

#### Pathologist accreditation

Participating pathologists were required to submit an anonymised synoptic report to the Pathology Assessment Committee (PAC) that was prepared in accordance with the Royal College of Pathologists of Australasia, Royal College of Pathologists (United Kingdom and Europe) or College of American Pathologists (United States of America) guidelines for reporting of colorectal carcinoma. Individual pathologists underwent workshop training and were provided with an instructional pathology manual detailing procedures required for standardised assessment of submitted specimens. Each surgeon and pathologist also reported three right hemicolectomy specimens to ensure understanding and compliance with procedures prior to commencement of the study. Pathologist at each centre were blinded as to treatment arm from which the operative specimens were obtained.

#### Surgical procedure

All robotic right hemicolectomies were undertaken with the da Vinci Xi® platform (Intuitive Surgical, Sunnyvale, CA, USA). The Xi is the dominant system in use throughout the world at the present time and therefore the system most suited to perform an international multicentre trial. The case numbers, surgeon experience and frequency of use of other platforms were not sufficient to include in a multicentre trial, nor draw conclusions about any colorectal procedure. Standard multiport laparoscopy was performed in the laparoscopic arm whilst hand assisted or other techniques such as single port were not permitted. In both arms, port positioning, order of procedural steps, type of anastomosis and need for conversion remained at the discretion of the surgeon. Due to the patient reported outcomes collected in this trial, every effort was made to maintain patient blinding. However, site data managers and outcome assessors were not blinded to their treatment allocation either pre- nor post-operatively.

### Safety monitoring

An independent data and data monitoring committee undertook six-monthly reviews of all reported adverse events and trial recruitment.

### Outcome measures

The primary objective was to determine if there is improvement in surgical morbidity after RRHC compared with LRHC. The primary outcome was surgical morbidity/mortality using the Comprehensive Complication Index (CCI) at 90 days, by randomised treatment arm [32–35]. The Clavien–Dindo (CD) classification [32] has been widely used to assess and stratify peri-operative complications, however the focus is only on the most severe complication. Events of lesser severity may not be reported, leading to an underestimation of the true overall post-operative morbidity. Therefore the CCI [32, 33] has been developed to summarise the entire post-operative experience of the patient. Although the CCI has been validated and could be potentially used as the primary endpoint in clinical trials, it does not account for other important factors in trials involving minimally invasive surgery such as conversion, complete tumour resection or unplanned readmission. As such, the CCI was used in this study to provide support to the concept of “successful surgery” as a potential primary endpoint. [36] The concept of “successful surgery” has been developed from a series of patient surveys and interviews using discrete questioning of objectives and based upon Parisi’s earlier prospective study [37] in which they defined “successful RRHC surgery” as meeting a series of predetermined surgical outcomes.

Secondary objectives were clinical and patient reported outcomes, including:

a) The pathological morphometric analysis of the submitted right hemicolectomy specimens, the plane of excision of mesocolon (mesocolic integrity), the completeness of mesocolic excision (CME) as assessed using the Benz Classification (30) and tumour staging according to the 8th edition of the AJCC cancer staging manual [38]. Central, blinded pathology review was planned for a later stage with an interim analysis, however with early study closure this was not performed. All pathology reporting was using a standardised reporting framework.

b) Patient-reported outcomes were measured using the European Organization for Research and Treatment of Cancer (EORTC) Quality of life- Core 30 (QLQ-C30) questionnaire, the EORTC colorectal cancer module QLQ-CR29, which collectively assess a range of symptoms and functional aspect relevant to colon cancer, and the EuroQOL 5 dimension- 5 level (EQ-5D-5L) [39–42] at baseline, discharge, one and three, six, 12, 18 and 24 months.

c) Pain was assessed by the Brief Pain Inventory (BPI) Short Form [43] on days one, three and five post-operatively.

d) Surgeon workload undertaken was assessed using the NASA Task Load Index (TLX) score of technical difficulty and cognitive load, which surgeons complete post-operatively [44, 45]. This is a multi-dimensional scale using a weighted averaging approach designed to obtain workload estimates from one or more operators while they are performing a task or immediately afterwards. Surgeons complete an assessment of the demands for six domains (mental, physical, temporal, performance, effort and frustration), which are then calculated to produce a final single numerical score, with higher scores representing a more difficult task.

e) Objective analysis of intraoperative surgical performance was done by the SAC through review of surgical case videos using Objective Clinical Human Reliability Analysis (OCHRA) and LCAT.

f) Assessment of intraoperative adverse events in order to report ‘near misses’ as measured by OCHRA and associated impact upon clinical outcomes.

g)’Successful surgical episode of care’ was prespecified in the statistical analysis plan to be assessed at 3 months rather than the originally planned 1 year, due to early study closure. It was defined by the following criteria:

- 1. Successfully completing the planned surgical approach without conversion.
- 2. Successful removal of tumour with no involved margins.
- 3. Successful post-operative course with no complications or deviation from the normal post-operative course and length of stay.
- 4. Successful post-discharge recovery with no readmissions or wound complications such as ventral hernia at laparotomy or specimen extraction site at 3 months.

### Statistical analysis

RoLaCaRT-1 was designed as an exploratory Phase II feasibility trial conducted with a prior plan to inform the design and assess the practicality of delivering a future definitive trial (RoLaCaRT-2) which will require more substantial time and resources. The study aimed to include 50 patients to provide an assessment of surgical morbidity/mortality, patient reported outcome and study feasibility. Analyses were conducted on a modified intention-to-treat (mITT) basis, including only patients who underwent surgery. The rationale for this was since RoLaCaRT-1 is a randomised Phase II trial the focus is on safety outcomes rather than efficacy. Intention-to-treat (ITT) analyses were also done as a sensitivity analysis.

Descriptive statistics reported are counts with percentages for categorical variables and mean (SD) or median (IQR) for continuous variables. Comparative analyses were performed using chi-squared tests for binary variables. Continuous variables were assessed using t-tests, or Wilcoxon rank–sum tests when normality assumptions were violated. For non-parametric estimation of the difference in medians between groups, the Hodges–Lehmann estimator was used to derive point estimates and corresponding 95% confidence intervals. The Mantel–Haenszel ordinal chi-square test was used to compare ordered categorical outcomes. Sensitivity analyses for the CCI outcome were performed using a log transformation (log(x + 1)) and linear regression, adjusting for stratification variables: sex, BMI (≤ 30 v > 30) and tumour site (caecum/ascending colon vs. hepatic flexure/proximal transverse colon). An additional sensitivity analysis was conducted that also incorporated surgeon/centre-level effects. Linear mixed-effects models were applied to account for potential clustering and evaluate the robustness of the findings, while acknowledging the limitations imposed by the small sample size. Patient reported outcome (PRO) questionnaire responses were scored according to their respective scoring manuals [39–42]. Generalised estimating equation (GEE) regression models were used to compare QLQ-C30 scale scores and the EQ-5D-5L VAS between trial arms, adjusted for baseline levels, age and sex. Due to issues with the distribution of the score for each QLQ-CR29 and EQ-5D-5L dimension, the proportions of participants reporting moderate to severe problems were compared to discharge, 30- and 90-days post-surgery.

All additional comparisons beyond the primary endpoint are considered exploratory. These analyses were conducted at a two-sided significance level of 5%, without adjustment for multiplicity. All statistical analyses used SAS software (version 9.4; SAS Institute Inc., Cary, NC, USA).

## Results

**Fig. 1:**
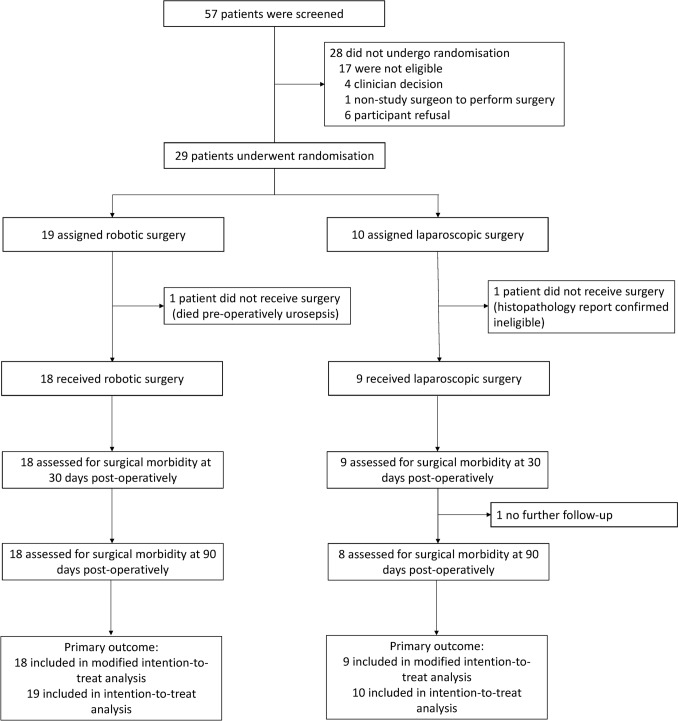
Consort diagram

Between August 2021 and November 2022, 29 participants were recruited from nine hospitals and randomised 2:1 to robotic (*n* = 19) and laparoscopic (*n* = 10). One participant randomised to LRHC was later deemed ineligible based on histopathology report. One participant randomised to a RRHC died of infection prior to surgery (Fig. 1). Fourteen surgeons from ten Australian sites and two UK were accredited. Baseline characteristics were well matched including for stratification factors with female 55%, mean age 71, BMI ≤ 30 55%, performance score 0 (52%) and caecal/ascending colon tumours 79% (Table 1). Of the 27 patients that underwent surgery all were alive at median follow-up of 6 months and free of recurrence assessed at a median follow-up of 4.8 months. The trial originally aimed to recruit 50 participants, but limited funding led to early closure after 15 months recruitment.

**Table 1:** Baseline characteristics of the study population according to treatment group

	Robotic (*n* = 19)	Laparoscopic (*n* = 10)
Age (years, mean [SD]) < 50 50–70 > 70	71.6 (12.7) 1 (5.3%) 7 (36.8%) 11 (57.9%)	70.6 (6.0) 0 7 (70.0%) 3 (30.0%)
Sex: Male Female	8 (42.1%) 11 (57.9%)	5 (50.0%) 5 (50.0%)
Height (cm, mean [SD])	164.0 (12.5)	166.8 (10.1)
Weight (kg, mean [SD])	81.4 (21.3)	81.5 (17.6)
BMI (kg/m^2^, mean [SD]) ≤ 30 > 30	30.1 (6.8) 10 (52.6%) 9 (47.4%)	29.1 (4.7) 6 (60.0%) 4 (40.0%)
Time since cancer diagnosis (weeks, mean [SD])	3.0 (2.0)	2.7 (1.6)
Time since colonoscopy (weeks, mean [SD])	2.9 (1.8)	3.3 (1.8)
Time since CT scan (weeks, mean [SD])	3.1 (1.9)	3.0 (2.4)
ECOG performance status:		
0	9 (47.4%)	6 (60.0%)
1	7 (36.8%)	4 (40.0%)
2	3 (15.8%)	0
Tumour site:		
Caecum/ascending colon	15 (78.9%)	8 (80.0%)
Hepatic flexure	3 (15.8%)	2 (20.0%)
Proximal transverse colon	1 (5.3%)	0
Abdominal girth (cm, mean [SD])	105.2 (18.4)	106.7 (22.9)
Tumour size–largest dimension on CT (cm, median [Q1, Q3])	3.9 (1.0, 4.8)	0.0 (0.0, 0.5)
Locoregional disease:		
Yes	8 (42.1%)	2 (22.2%)
No	11 (57.9%)	7 (77.8%)
Missing		1
Distant metastases:		
Yes	0	0
No	19 (100.0%)	9 (100.0%)
Missing		1
CEA (ug/L, median [Q1, Q3])	1.8 (1.2, 4.0)	1.5 (1.3, 3.3)

Data are *n* (%) unless otherwise indicated

### Surgical and pathology outcomes

Ten surgeons performed surgery on 27 participants who all received their assigned surgery. Two surgeons each performed seven operations (one at two different centers), one surgeon performed 3 operations, 3 surgeons performed 2 each and the remaining four surgeons each carried out 1 operation. There was no operative mortality, and no intraoperative adverse events or significant haemorrhage events reported. No conversions occurred nor any pre-operative intra- or extra-corporeal anastomosis plans required alteration. All patients in the robotic group (18/18, 100%) underwent intracorporeal anastomosis. Within the laparoscopic group, two patients (22.2%) received intracorporeal anastomosis, while the remaining seven (77.8%) underwent extracorporeal anastomosis. The mean operative time was increased in the robotic group (mean 226 vs. 187 min, *p* = 0.03) ***(***Table 2). All had R0 resection. The CME rate (Benz classification Type 0) was 53% in the robotic group vs. 50% in the laparoscopic group and the integrity of the mesocolon (West classification) was 94 v 100% as there was a defect in the posterior mesocolon in 1 robotic case (Table 3). Mean lymph node yield was higher in the robotic group than in the laparoscopic group, although the difference was not statistically significant (25.2 vs 19.1). All reported adverse events were reviewed by the trial executive team for completeness and accuracy, and reporting investigators were asked to verify their submissions. Each event was then assessed against predefined grading criteria prior to analysis.

**Table 2:** Surgical overview and peri-operative recovery according to treatment group

	Robotic (*n* = 19)	Laparoscopic (*n* = 10)
*Surgical overview:*		
Received surgery	18/19 (94.7%)	9/10 (90.0%)
ASA Classification at time of operation:		
A patient with mild systemic disease	7 (38.9%)	5 (55.6%)
A patient with severe systemic disease	11 (61.1%)	4 (44.4%)
Patient had prior abdominal surgery?	11/18 (61.1%)	3/9 (33.3%)
Mechanical bowel preparation used	14/18 (77.8%)	6/9 (66.7%)
Received prophylactic antibiotics at time of surgery	18/18 (100.0%)	9/9 (100.0%)
Surgical assistants	18/18 (100.0%)	9/9 (100.0%)
Planned anastomosis:		
Intracorporeal anastomosis	18 (100.0%)	2 (22.2%)
Extracorporeal anastomosis	0	7 (77.8%)
Type of anastomosis performed:		
Intracorporeal anastomosis	18 (100.0%)	2 (22.2%)
Extracorporeal anastomosis	0	7 (77.8%)
Anastomosis converted	0/18	0/9
Extraction site:		
Upper midline	0	3 (33.3%)
Periumbilical midline	0	5 (55.6%)
Pfannenstiel	9 (50.0%)	1 (11.1%)
Right or left iliac fossa muscle split	9 (50.0%)	0
Final extraction incision length (cm)	5.7 (1.5)	5.4 (1.8)
Total estimated blood loss (ml)	40.3 (44.7)	69.7 (90.0)
Total number of laparoscopic ports	1.4 (1.6)	3.8 (0.8)
Total number of robotic ports		
3	1 (5.6%)	
4	16 (88.9%)	
5	1 (5.6%)	
Procedure at start of operation:		
Robotic	18 (100.0%)	
Laparoscopic		9 (100.0%)
Robotic/laparoscopic resection converted	0/18	0/9
Clinical curative resection	18/18 (100.0%)	9/9 (100.0%)
Time in operating theatre (min)	226 (46)^*^	187 (35)^*^
Time from first incision to wound closure completion (min)	172 (33)^†^	124 (34)^†^
Robotic surgery: Time from moving cart to bedside to leaving console (min)	135 (39)	
*Peri-operative recovery:*		
Number of days requiring parenteral narcotics	*n* = 18; 1.7 (2.0)	*n* = 9; 1.2 (0.8)
Number of days receiving oral analgesics	*n* = 18; 4.1 (2.0)^*^	*n* = 9; 9.6 (8.9)^*^
Post-operative antibiotics administered	5/18 (27.8%)	3/9 (33.3%)
Days post-operative antibiotics administered	*n* = 5; 3.8 (3.6)	*n* = 3; 8.0 (4.6)
Thromboprophylaxis medication administered	17/18 (94.4%)^†^	4/9 (44.4%)^†^
Days thromboprophylaxis medication administered	*n* = 17; 8.4 (12.1)	*n* = 4; 18.5 (12.0)
Number of days post-op before first flatus	*n* = 16; 1.8 (0.7)	*n* = 7; 2.4 (1.3)
Number of days post-op before first bowel movement	n = 18; 2.8 (1.0)	*n* = 9; 2.8 (1.4)
Number of days before diet tolerance (commencement of solid diet)	*n* = 17; 2.4 (2.7)	*n* = 8; 3.3 (2.5)
Nasogastric tube inserted	3/18 (16.7%)	2/9 (22.2%)
Length of hospital stay (days)	*n* = 18, 4.0 (3.0, 6.0)	*n* = 9, 6.0 (4.0, 8.0)
≥ 1 day in ICU	0/18^*^	2/9 (22.2%)^*^
Re-operation	0/18	0/9

Data are n/N (%), mean (SD) or median (IQR)

Significance levels for comparison of procedure time and peri-operative recovery variables between Robotic and Laparoscopic groups: *P < 0.05; †*P* < 0.01

**Table 3:** Surgical and pathology^*^ outcomes according to treatment group

	Robotic (*n* = 17)	Laparoscopic (*n* = 8)
Length of resected large intestine (mm)	234.3 (66.0)	217.0 (47.5)
Surface area of mesentery (cm^2^)^†^	143.3 (69.1)	132.4 (60.0)
Distance from tumour to high vascular tie (mm)	134.8 (31.9)	142.3 (44.2)
Distance from colon wall to high vascular tie (mm)	104.2 (20.3)	111.3 (35.1)
Distance to posterior mesocolic margin (mm)	33.1 (29.2)	35.1 (19.0)
Distance of tumour to distal resection margin (mm)	137.9 (66.0)	99.4 (32.4)
Distal resected margin ≥ 5cm	17 (100%)	7 (88%)
Lymph node yield	25.2 (13.3)	19.1 (4.5)
Positive lymph nodes	7 (41%)	2 (25%)
Number of positive lymph nodes:		
Missing	1	1
0	10 (58.8%)	6 (75.0%)
1	4 (23.5%)	2 (25.0%)
2	2 (11.8%)	
3	1 (5.9%)	
R0 resection rate (Completeness of excision)	17 (100.0%)	8 (100%)
Completeness of mesocolon:		
Missing	1	1
Type 0	9 (52.9%)	4 (50.0%)
Type 1	3 (17.6%)	2 (25.0%)
Type 2	5 (29.4%)	1 (12.5%)
Type 3		1 (12.5%)
Integrity of mesocolon:		
A – Intact mesocolon: mesocolic plane	16 (94%)	8 (100%)
B – Laceration in the mesocolon: intramesocolic plane	1 (6%)	
Pathologic Staging (TNM)		
T Stage (1–4)		
Tumour not seen (Tx)		1 (11.1%)
Invades submucosa (T1)	1 (5.6%)	2 (22.2%)
Invades muscularis propria (T2)	3 (16.7%)	1 (11.1%)
Beyond muscularis propria (T3)	10 (55.6%)	2 (22.2%)
Tumour spread into peritoneum (T4a)	4 (22.2%)	3 (33.3%)
N Stage (0 +)		
No visible nodes or smooth bordered node = N0	11 (61.1%)	6 (66.7%)
1 node = N1a	4 (22.2%)	3 (33.3%)
2–3 nodes = N1b	3 (16.7%)	
M Stage		
M0	4 (22.2%)	3 (33.3%)
X–not stated	14 (77.8%)	6 (66.7%)

Data are *n* (%) or mean (SD)

^*^Pathology was not available for 1 patient in each arm that received surgery. *P* > 0.05 for all comparisons

^†^2 missing values in Robotic arm

**Fig. 2:**
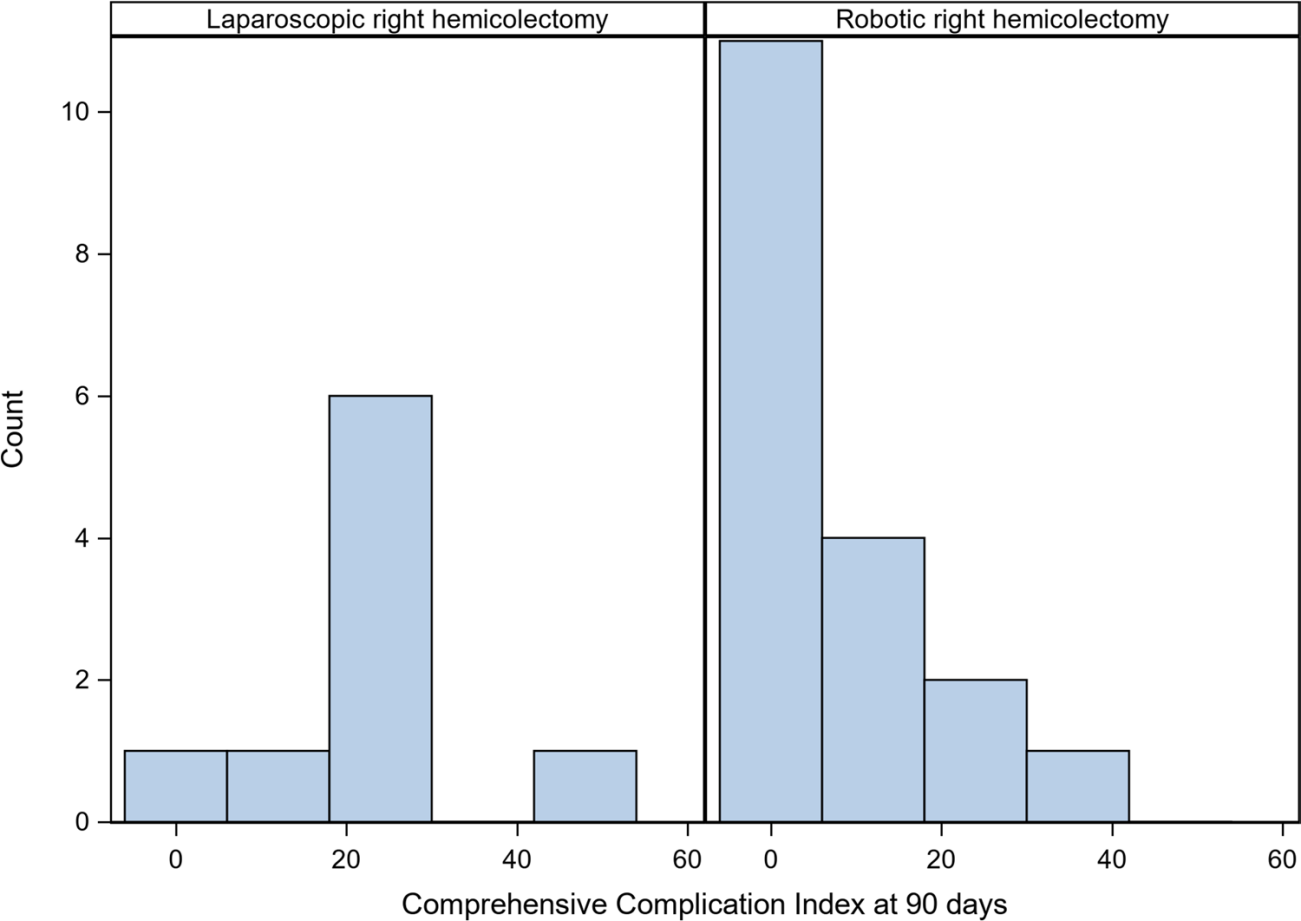
Frequency distribution of the Comprehensive Complication Index scores at 90 days (mITT population)

Figure 2 presents the distribution of CCI scores at 90 days for patients who underwent surgery. In the mITT analysis CCI scores were lower for patients that underwent RRHC compared to LRHC at 30 and 90 days (median (Q1, Q3): 0 (0, 9) vs. 21 (21, 26); median difference: 21; 95% CI 9–26; p = 0.005 at 30 days; *p* = 0.008 at 90 days). In the ITT analysis the difference in scores between groups was smaller (median difference: 14; 95% CI 0–21) and more uncertain, as the confidence interval included zero. However, the tests for group differences remained statistically significant (Table 4). In the sensitivity analysis (Supplementary Table 1), adjusting for stratification factors and clustering by surgeon and centre, the geometric mean of expected 90-day CCI scores (+ 1) in the laparoscopic group was 4.9 (95% CI 1.7–14.6, *p* = 0.0078) times higher than in the robotic group. At 30 days the ratio was 5.1 (95% CI 1.8–14.8; *p* = 0.0059). In the ITT analysis differences were smaller however statistically significant.

**Table 4:** Adverse events: Comprehensive Complication Index (CCI) at 30 and 90 days and worst complication grade suffered across all early and late complications up to 90 days according to treatment group

	Robotic		Laparoscopic		Laparoscopic—Robotic	
	N	Median [Q1, Q3]	*n*	Median [Q1, Q3]	Difference (95% CI)^*^	P^*^
Modified ITT population:						
CCI score at 30 days	18	0.0 (0.0, 8.7)	9	20.9 (20.9, 26.2)	20.9 (8.7–26.2)	0.005
CCI score at 90 days^†^	18	0.0 (0.0, 8.7)	9	20.9 (20.9, 26.2)	20.9 (8.7–26.2)	0.008
ITT population^‡^:						P
CCI score at 30 days	19	0.0 (0.0, 12.2)	10	20.9 (8.7, 26.2)	14.0 (0.0–20.9)	0.028
CCI score at 90 days^†^	19	0.0 (0.0, 12.2)	10	20.9 (8.7, 26.2)	14.0 (0.0–20.9)	0.038
Worst grade across all complications (mITT):	Robotic (*n* = 18)		Laparoscopic (*n* = 9)			P^**^
	*n* (%)		*n* (%)			
0	11 (61.1)		1 (11.1)			0.003
1	4 (22.2)		1 (11.1)			
2	2 (11.1)		4 (44.4)			
3–a	1 (5.6)		3 (33.3)			

*mITT* modified intention-to-treat

^*^Hodges-Lehmann estimate and 95% confidence interval for location shift. Wilcoxon rank-sum test

^†^1 patient in the Laparoscopic arm who suffered grade 3 complications in the first 30 days did not have follow-up to 90 days. This patient’s 90-day score is imputed from the 30-day score

^‡^The patient that died of infection prior to surgery was assigned a score of 100 as per the CCI scoring instructions. The ineligible patient was assigned a score of zero

^**^Mantel–Haenszel ordinal chi-square test

In a post-hoc analysis the most severe post-operative complication suffered by a patient up to 90 days was higher in grade after laparoscopic surgery compared with robotic surgery. The worst complication was grade I for 4 (22%) patients in the robotic arm vs 1 (11%) in the laparoscopic arm, grade II for 2 (11%) vs 4 (44%), respectively and grade III-a for 1 (6%) vs 3 (33%) (*p* = 0.003**,** Table 4). There were no grade IV or higher adverse events. A detailed summary of all complications by Clavien-Dindo grade is provided in Table 5. Two patients that underwent laparoscopic surgery experienced an anastomotic leak, one grade II and one grade IIIa. There were three ileus complications in the robotic group (two grade I and one grade II) and two grade II in the laparoscopic group. One grade II surgical site infection was observed in the robotic group. No patient required re-operation.

**Table 5:** Types of post-operative adverse events to 30 and 90 days and severity score by the Clavien–Dindo classification

	Robotic			Laparoscopic		
Clavien-Dindo classification (grade)	I	II	III-a	I	II	III-a
*Early complications (up to 30 days post-surgery) – number assessed*	*n* = 18			*n* = 9		
Intra-operative injury	0	0	0	0	0	0
Ileus	2 (11.1%)	1 (5.6%)	0	0	2 (22.2%)	0
Anastomotic leak	0	0	0	0	1 (11.1%)	1 (11.1%)
Anastomotic stricture	0	0	0	0	0	0
Surgical site infection	0	1 (5.6%)	0	0	0	0
Venous thromboeobolism	0	0	0	0	0	0
Acute renal failure	0	0	0	0	0	0
Ischaemic heart disease	0	0	0	0	0	0
Respiratory distress	0	0	0	0	0	0
Aspiration	0	0	0	0	0	0
Respiratory complications	0	0	0	0	1 (11.1%)	0
Haematoma	2 (11.1%)	0	0	0	0	1^*^ (11.1%)
Gastrointestinal perforation	0	0	0	0	0	0
Wound dehiscence	0	0	0	0	1^*^ (11.1%)	0
Fever	0	0	0	0	0	1 (11.1%)
Urinary retention	0	0	0	0	0	0
Urinary tract infection	0	1 (5.6%)	0	0	0	0
Intra-abdominal infection	0	0	0	0	0	1 (11.1%)
Hemorrhage/bleeding associated with surgery, intra-operative or post-operative	1 (5.6%)	0	0	0	1 (11.1%)	1 (11.1%)
Intestinal obstruction	0	0	0	0	0	0
Other complications	1 (5.6%)	0	0	1 (11.1%)	0	0
*Late complications (post 30 days to 90 days) – number assessed*	*n* = 18			*n* = 8		
Gastrointestinal fistula	0	0	0	0	0	0
Anastomotic stenosis	0	0	0	0	0	0
Anastomotic stricture	0	0	0	0	0	0
Intestinal obstruction	0	0	0	0	0	0
Internal hernia	0	0	0	0	0	0
Wound hernia at port	0	0	0	0	0	0
Wound hernia at surgery site	0	0	0	0	0	0
Other	0	0	1 (5.6%)	0	0	0

^*^Resulted in readmission to hospital

### Episodes of ‘successful surgery’

Of the four components that comprise ‘successful surgical episode of care’, there was no difference between the groups with regards to i) conversion, and ii) clear margins and good quality specimen. For the third component-iii) readmissions or wound complications such as ventral hernia at laparotomy or specimen extraction site, one additional readmission occurred in the laparoscopic arm (2 (22.2%) vs 1 (5.6%)), though no wound complications were reported in either group. The rate of the final component iv) complications or deviation from the normal post-operative course and length of stay, was lower in the robotic arm (4 (22.2%) vs 5 (55.6%)). Although 3 of the 4 components did not show marked differences, the combined overall rate of ‘successful surgical episodes of care’ was significantly greater for RRHC compared to LRHC at 90 days (13/18 (72.2%) v. 2/9 (22.2%), *P* = 0.014; Table 6).

**Table 6:** Successful surgical episode of care to 90 days according to treatment group

	Robotic (*n* = 18) *n* (%)	Laparoscopic (*n* = 9) *n* (%)	P-value^*^
Conversions	0	0	
Clear margins and good quality specimen	18	9	
Readmission	1	2	
Wound complication	0	0	
Complications or deviation from normal post-operative course and length of stay^†^ - Ileus or insertion of nasogastric tube - Anastomotic complication - Surgical site infection - Prolonged hospital stay	4^‡^ 4 0 1 2	5^§^ 2 2 0 3	
Successful surgical episode of care	13 (72.2%)	2 (22.2%)	0.014

^*^Chi-squared test

^†^Prolonged hospital stay defined as length of stay > 7 days

^‡^1 patient met 3 of the criteria and 1 other met 2 of the criteria

^§^1 patient met 3 of the criteria

### Patient reported outcomes

A total of 25 participants were included in the repeated measures analysis of QLQ-C30 domains, having completed baseline and a minimum of one post-surgical time point. Mean scores between groups were similar for all functional domains and global quality of life at all assessed time points. The robotic group reported lower symptom burden for nausea/vomiting at 90 days (mean 3.7 vs 12.5; adjusted difference = − 13.8 [95% CI − 21.1-− 6.5]) and appetite loss at 30 days (9.3 vs 28.6; − 27.4 [95% CI − 50.8-− 3.9]); which are considered small and medium sized differences, respectively, according to the EORTC guidelines [46]. EQ-5D-5L VAS and BPI severity or interference scores were similar between groups at days 1, 3 or 5 (Supplementary Tables 2–6).

### Assessment of surgeons workload performance

NASA-TLX was completed for all 27 patients that underwent surgery. The overall weighted workload scores were 16 points lower following robotic surgery indicating lower perceived workload compared to laparoscopic however this difference was not significant (mean 31 vs 47; difference: -16; [95% CI -32–1], *p* = 0.065). Of the individual domains, load ratings in the robotic group were significantly lower for physical demand (difference -28 [95% CI -43- -12], *p* = 0.001) and performance (-21 [95% CI -40- -2], *p* = 0.03), with no difference being demonstrated in the domains of mental demand, temporal demand, effort or frustration. It is worth noting that the surgeons weighted the dimensions mental demand, performance and effort highest in their contribution to the overall workload (Supplementary Table 7).

## Discussion

This was the first pilot multinational randomised trial of robotic versus laparoscopic surgery for cancer of the right colon comparing the overall complication rate, or CCI, as the primary end point. To ensure the highest level of quality control that is so important in a surgical trial, strict accreditation criteria for both surgeon and pathologist eligibility were used, resulting in no conversions and R0 clearance for all patients, in both arms of the study. Previous large studies have demonstrated non-inferior oncological outcomes when comparing open with laparoscopic surgery for cancer of the colon [47, 48]. These studies have also demonstrated lower complication rates especially in the elderly [49]. This has led to widespread adoption of minimally invasive approaches for the treatment of colon cancer. With the more recent introduction of robotic approaches there has been attempts at direct comparison with laparoscopic surgery [50, 51]. These have largely been based on length of stay and conversion. Given that both techniques are minimally invasive, it is therefore not unexpected that outcomes were shown to be similar but somewhat more expensive for robotic approaches [52]. Similarly, oncological outcomes would be expected to be similar assuming the same oncological principles have been followed. Without supportive evidence there would seem to be no explanation for the rapid rise in the use of robotics for the treatment of colon cancer. In order to justify the use of robotics and associated expenses we need to examine for potential advantages for robotics or otherwise, not based on traditional trial outcomes. This led to the trial design adopted in this study.

The comprehensive complication index has previously been proposed and validated as a more sensitive end point for assessing surgical outcomes and potentially reducing sample sizes in randomised control trials [33]. RoLaCaRT-1 was designed as a pilot study to explore this approach and directly assist the design of larger follow-on studies which would be expected to require extensive financial and resource support across many years. Study funders rightly increasingly request pilot data to ensure delivery is achievable and the methodology is optimised before commencing resource heavy, long-term studies. Our experience and data from multiple outcome assessments informs such decisions and supports our focus on morbidity and use of the CCI. Accredited surgeons are shown to deliver good quality right colon specimens with either minimally invasive approach, so this traditional endpoint no longer appears appropriate.

Significant advances in imaging, evidence-based use of neoadjuvant therapies and precision surgery have led to improved outcomes for rectal cancer. Overall survival for patients with cancers in the right colon has been reported to be lower compared with tumours of other parts of the colon and rectum [53, 54]. This has been traditionally attributed to a more advanced stage at presentation due to delayed diagnosis and potentially the underlying cancer biology. However, operative performance in terms of oncological clearance as expressed by higher lymph nodes harvested was shown to eliminate this difference in survival rate [52]. This may be because a higher yield could lead to stage migration and consequently to more intensive systemic therapy, associated with a better prognosis. This would suggest that there may be a need to improve the quality of surgery and the resection. This may be achieved with complete mesocolic excision and possibly further enhanced with intracorporeal anastomosis. However, there has been reluctance in the surgical community for widespread adoption of these techniques. It is not clear whether this reluctance is due to increased surgical difficulty or if further evidence is required to demonstrate advantages in both short and long-term outcomes.

This pilot study has demonstrated a significant reduction in the overall morbidity for patients undergoing minimally invasive right colectomy for cancer when the operation is performed using robotics. If the reduced overall morbidity seen here in this pilot was confirmed in a larger study, there is a potential for earlier discharge, return to normal function and, where indicated, allow timely adjuvant chemotherapy. Patients reported reduced nausea and vomiting in the RRHC group possibly as a robotic approach is associated with higher rates of intra corporeal anastomosis with reduced tissue traction and ileus rates as demonstrated in the MIRCAST observational study [55], although results should be interpreted with caution due to the sample size. CME surgery results in larger mesenteric area resection, better quality specimen and higher number of harvested lymph nodes but is acknowledged as a technically demanding operation [56]. Despite the study mean BMI being nearly 30 kg/m^2^ a similar rate of CME was performed in both groups with no differences seen in overall pathology specimen quality grading or completeness of the resection. CME can be associated with increased morbidity. However, in this trial with experienced surgeons no such findings were observed with no reported incidence of intraoperative vascular injuries, post-operative bleeding or chyle leaks. In keeping with prior reports, the operating times were longer in the RRHC group as compared to the LRHC as demonstrated by the previous studies [14].

Whilst the CCI aims to provide overall cumulative burden of post-operative complications as a measurement of the entire post-operative experience of the patient there are a number of other factors and outcomes deemed important by the patient as part of the overall surgical experience. As such, the concept of a “successful episode of surgery” has been proposed with specific reference to minimally invasive surgery of the colon as being the CCI in combination with non-conversion, clear margins and no incisional hernia. Whilst the results in this pilot study with a higher rate of ‘successful surgery’ after RRHC, this appears to be primarily driven by the difference in complications. Whilst this clearly needs further study and external validation, our data suggests “successful surgery” is a reasonable outcome to use as a primary endpoint and particularly where robotics and laparoscopy are being compared. The observed differences highlight a potentially large effect size and can directly inform sample size calculations for larger, adequately powered studies.

There has been increased awareness of the physical and mental stress that surgeons experience throughout their career. A robotic approach has been thought to reduce some of the physical demands of laparoscopy by improving ergonomics and working position of the operating surgeon during long cases [57]. Using a widely used and validated measure, the NASA-TLX tool, surgeon reported data in this pilot study supports robot use in right hemicolectomy procedures which we consider likely to be generalizable to other major abdominal procedures. Assessment of surgeon cognitive load was lower during robotic compared with laparoscopic surgery.

### Limitations

There are limitations to this study. Firstly, patient numbers are small and the study closed early due to limited funding. Nevertheless, we demonstrated that multicentre, multinational recruitment into robotic vs. laparoscopic studies is possible and note a difference in the primary outcome can be demonstrated. It can be argued that the complication rates of the laparoscopic control group are higher than expected, even when performed by accredited surgeons. We acknowledge the comparisons presented are unpowered for secondary outcomes and as such should be interpreted with caution. Secondly, due to the nature of the study, surgeon blinding was not possible. This may have led to differences in post-operative care depending on the operative approach taken. Cost-analysis was not included in this pilot study, and it is possible that the longer operating time, cost of consumables and infrastructure in the robotic arm may balance, or possibly out-weigh the interpretation of morbidity gains. A cost-effectiveness study is planned within a future larger phase III study, RoLaCaRT 2.

## Conclusion

This pilot study suggests that a robotic approach for right hemicolectomy may have improved short-term surgical morbidity, surgeon physical demand and performance with similar specimen quality and patient reported outcomes when compared to laparoscopic surgery for right sided colon cancer. Larger studies with a focus on morbidity are planned to confirm or refute these findings.

## Supplementary Information

Below is the link to the electronic supplementary material.

Supplementary file1 (DOCX 93 KB)

## Data Availability

The data that support the findings of this study are stored securely at the University of Sydney and may be available from the corresponding author upon reasonable request. Access to the data will require approval from the RoLaCaRT Trial Management Executive Committee and a University of Sydney data sharing agreement.
