# Supplementary material for: RoLaCaRT-1: pilot randomised phase II study of robotic vs laparoscopic hemicolectomy for right colon cancer
Source: Surg Endosc. 2025 Dec 10;40(3):2062–77. doi: 10.1007/s00464-025-12400-1 (PMC12971845; doi:10.1007/s00464-025-12400-1)
Supplement: Supplementary file 1 — Supplementary file1 (DOCX 93 KB) [file 464_2025_12400_MOESM1_ESM.docx]

**Supplementary Table 1: Sensitivity analysis – Multivariable linear regression analysis of log-transformed CCI Score (log(x+1)) with adjustment for stratification factors**

| *Analysis* | *Laparoscopic - Robotic*  *Difference (95% CI)* | *SE* | *Ratio (95% CI)* | *P-value* |
| --- | --- | --- | --- | --- |
| *mITT analysis* |  |  |  |  |
| Model 1: Linear regression of the 30-day CCI score adjusted for sex, BMI and tumor site | 1.82 (0.71 - 2.93) | 0.54 | 6.17 (2.03 - 18.80) | 0.0026 |
| Model 2: Linear mixed-effects model of the 30-day CCI score adjusted for sex, BMI, tumor site and surgeon/center | 1.63 (0.57 - 2.69) | 0.49 | 5.11 (1.76 - 14.79) | 0.0059 |
| Model 3: Linear regression of the 90-day CCI score adjusted for sex, BMI and tumor site | 1.80 (0.66 - 2.94) | 0.55 | 6.05 (1.93 - 18.97) | 0.0035 |
| Model 4: Linear mixed-effects model of the 90-day CCI score adjusted for sex, BMI, tumor site and surgeon/center | 1.59 (0.50 - 2.68) | 0.50 | 4.92 (1.66 - 14.63) | 0.0078 |
| *ITT analysis* |  |  |  |  |
| Model 5: Linear regression of the 30-day CCI score adjusted for sex, BMI and tumor site | 1.38 (0.14 - 2.62) | 0.60 | 3.99 (1.16 - 13.75) | 0.030 |
| Model 6: Linear mixed-effects model of the 30-day CCI score adjusted for sex, BMI, tumor site and surgeon/center | 1.21 (0.07 - 2.35) | 0.53 | 3.36 (1.08 - 10.47) | 0.039 |
| Model 7: Linear regression of the 90-day CCI score adjusted for sex, BMI and tumor site | 1.37 (0.11 - 2.63) | 0.61 | 3.93 (1.12 - 13.81) | 0.034 |
| Model 8: Linear mixed-effects model of the 90-day CCI score adjusted for sex, BMI, tumor site and surgeon/center | 1.18 (0.03 - 2.34) | 0.54 | 3.27 (1.03 - 10.38) | 0.045 |

**Supplementary Table 2: QLQC30 domains scores^*^ over time**

|  | **Robotic** | | **Laparoscopic** | | **Robotic - Laparoscopic** | |
| --- | --- | --- | --- | --- | --- | --- |
| **Domain/Time-point** | **N** | **Mean (SD)** | **N** | **Mean (SD)** | **Adjusted Difference (95% CI)^†^** | **P^†^** |
| D1: Physical Functioning |  |  |  |  |  |  |
| Baseline | 18 | 84.8 (18.8) | 7 | 91.0 (12.9) |  |  |
| Discharge | 15 | 60.9 (22.2) | 5 | 60.0 (32.3) | 1.7 (-25.9 - 29.3) | 0.903 |
| 30 days post-operative | 18 | 80.7 (16.6) | 7 | 74.0 (21.8) | 8.7 (-6.7 - 24.2) | 0.266 |
| 90 days post-operative | 18 | 83.9 (20.2) | 8 | 81.7 (11.7) | 1.2 (-8.6 - 10.9) | 0.812 |
| D2: Role Functioning |  |  |  |  |  |  |
| Baseline | 18 | 88.0 (25.4) | 7 | 85.7 (26.2) |  |  |
| Discharge | 15 | 37.8 (35.3) | 5 | 46.7 (44.7) | -12.2 (-51.3 - 26.8) | 0.539 |
| 30 days post-operative | 18 | 76.9 (28.1) | 7 | 52.4 (42.4) | 21.6 (-8.8 - 51.9) | 0.164 |
| 90 days post-operative | 18 | 82.4 (32.6) | 8 | 77.1 (30.8) | -6.2 (-27.5 - 15.1) | 0.567 |
| D3: Emotional Functioning |  |  |  |  |  |  |
| Baseline | 18 | 69.4 (27.3) | 7 | 81.0 (15.7) |  |  |
| Discharge | 15 | 75.6 (23.7) | 5 | 66.7 (25.7) | 13.4 (-8.3 - 35.1) | 0.227 |
| 30 days post-operative | 18 | 83.8 (21.7) | 7 | 83.3 (14.4) | 6.2 (-8.0 - 20.4) | 0.392 |
| 90 days post-operative | 18 | 77.3 (22.3) | 8 | 78.1 (22.2) | 8.0 (-8.0 - 24.1) | 0.325 |
| D4: Cognitive Functioning |  |  |  |  |  |  |
| Baseline | 18 | 74.1 (16.4) | 7 | 88.1 (12.6) |  |  |
| Discharge | 15 | 73.3 (29.4) | 5 | 73.3 (25.3) | 15.1 (-9.9 - 40.2) | 0.236 |
| 30 days post-operative | 18 | 80.6 (22.3) | 7 | 78.6 (24.9) | 11.8 (-12.2 - 35.8) | 0.334 |
| 90 days post-operative | 18 | 70.4 (28.3) | 8 | 72.9 (28.1) | 11.2 (-10.1 - 32.4) | 0.303 |
| D5: Social Functioning |  |  |  |  |  |  |
| Baseline | 18 | 88.0 (24.8) | 7 | 83.3 (19.2) |  |  |
| Discharge | 15 | 50.0 (36.7) | 5 | 70.0 (44.7) | -22.5 (-63.2 - 18.2) | 0.278 |
| 30 days post-operative | 18 | 81.5 (24.8) | 7 | 73.8 (21.2) | 5.7 (-10.6 - 21.9) | 0.495 |
| 90 days post-operative | 17 | 84.3 (20.0) | 8 | 79.2 (21.4) | 1.5 (-16.9 - 19.9) | 0.872 |
| D6: Global health status/QoL | |  |  |  |  |  |
| Baseline | 18 | 69.0 (17.3) | 7 | 79.8 (16.6) |  |  |
| Discharge | 15 | 56.7 (21.2) | 5 | 60.0 (24.6) | 1.2 (-16.5 - 19.0) | 0.893 |
| 30 days post-operative | 18 | 71.3 (19.4) | 7 | 71.4 (19.2) | 4.1 (-11.7 - 20.0) | 0.610 |
| 90 days post-operative | 18 | 68.5 (18.2) | 8 | 68.8 (21.2) | 3.7 (-11.4 - 18.9) | 0.628 |
| D7: Fatigue |  |  |  |  |  |  |
| Baseline | 18 | 24.7 (26.8) | 7 | 23.8 (20.7) |  |  |
| Discharge | 15 | 51.9 (26.4) | 5 | 53.3 (33.7) | -4.7 (-33.8 - 24.3) | 0.749 |
| 30 days post-operative | 18 | 26.5 (17.9) | 7 | 36.5 (27.8) | -11.3 (-31.6 - 9.0) | 0.276 |
| 90 days post-operative | 18 | 27.2 (27.8) | 8 | 30.6 (19.5) | -7.5 (-24.4 - 9.5) | 0.387 |
| D8: Nausea / Vomiting |  |  |  |  |  |  |
| Baseline | 18 | 5.6 (12.8) | 7 | 2.4 (6.3) |  |  |
| Discharge | 15 | 14.4 (20.8) | 5 | 26.7 (30.3) | -15.9 (-42.1 - 10.3) | 0.235 |
| 30 days post-operative | 18 | 3.7 (9.1) | 7 | 4.8 (8.1) | -4.3 (-9.4 - 0.9) | 0.103 |
| 90 days post-operative | 18 | 3.7 (9.1) | 8 | 12.5 (11.8) | -13.8 (-21.1 - -6.5) | <.001 |
| D9: Pain |  |  |  |  |  |  |
| Baseline | 18 | 21.3 (32.2) | 7 | 23.8 (25.2) |  |  |
| Discharge | 15 | 45.6 (39.1) | 5 | 36.7 (38.0) | 11.4 (-22.0 - 44.9) | 0.503 |
| 30 days post-operative | 18 | 18.5 (26.1) | 7 | 21.4 (23.0) | -2.9 (-18.9 - 13.1) | 0.721 |
| 90 days post-operative | 18 | 17.6 (30.0) | 8 | 18.8 (13.9) | 0.9 (-14.3 - 16.1) | 0.906 |
| D10: Dyspnoea |  |  |  |  |  |  |
| Baseline | 18 | 13.0 (25.9) | 7 | 4.8 (12.6) |  |  |
| Discharge | 15 | 22.2 (30.0) | 5 | 6.7 (14.9) | 11.7 (-6.2 - 29.6) | 0.201 |
| 30 days post-operative | 18 | 7.4 (14.3) | 7 | 4.8 (12.6) | 1.5 (-9.1 - 12.2) | 0.782 |
| 90 days post-operative | 18 | 3.7 (15.7) | 8 | 16.7 (25.2) | -16.5 (-34.9 - 1.9) | 0.079 |
| D11: Insomnia |  |  |  |  |  |  |
| Baseline | 18 | 33.3 (37.9) | 7 | 28.6 (23.0) |  |  |
| Discharge | 15 | 37.8 (35.3) | 5 | 40.0 (14.9) | -11.0 (-35.6 - 13.6) | 0.379 |
| 30 days post-operative | 18 | 24.1 (29.8) | 7 | 38.1 (35.6) | -20.0 (-41.6 - 1.5) | 0.069 |
| 90 days post-operative | 18 | 22.2 (30.2) | 8 | 33.3 (30.9) | -17.1 (-41.2 - 6.9) | 0.163 |
| D12: Appetite loss |  |  |  |  |  |  |
| Baseline | 18 | 13.0 (25.9) | 7 | 4.8 (12.6) |  |  |
| Discharge | 15 | 35.6 (32.0) | 5 | 40.0 (36.5) | -16.0 (-49.9 - 18.0) | 0.357 |
| 30 days post-operative | 18 | 9.3 (25.1) | 7 | 28.6 (35.6) | -27.4 (-50.8 - -3.9) | 0.022 |
| 90 days post-operative | 18 | 11.1 (28.0) | 8 | 12.5 (24.8) | -11.2 (-28.5 - 6.1) | 0.204 |
| D13: Constipation |  |  |  |  |  |  |
| Baseline | 18 | 13.0 (20.3) | 7 | 19.0 (17.8) |  |  |
| Discharge | 15 | 20.0 (32.9) | 5 | 26.7 (27.9) | -5.4 (-34.4 - 23.6) | 0.715 |
| 30 days post-operative | 18 | 13.0 (20.3) | 7 | 14.3 (17.8) | -2.3 (-17.3 - 12.7) | 0.765 |
| 90 days post-operative | 18 | 11.1 (19.8) | 8 | 12.5 (17.3) | 0.6 (-14.2 - 15.5) | 0.935 |
| D14: Diarrhoea |  |  |  |  |  |  |
| Baseline | 18 | 16.7 (32.8) | 7 | 14.3 (26.2) |  |  |
| Discharge | 15 | 35.6 (40.8) | 5 | 33.3 (23.6) | 6.0 (-20.5 - 32.5) | 0.657 |
| 30 days post-operative | 18 | 13.0 (23.3) | 7 | 23.8 (25.2) | -7.2 (-26.8 - 12.4) | 0.470 |
| 90 days post-operative | 18 | 20.4 (30.5) | 8 | 8.3 (15.4) | 14.5 (-3.9 - 32.8) | 0.122 |
| D15: Financial Problems |  |  |  |  |  |  |
| Baseline | 18 | 0.0 (0.0) | 7 | 0.0 (0.0) |  |  |
| Discharge | 15 | 13.3 (27.6) | 5 | 13.3 (29.8) | 3.7 (-20.9 - 28.4) | 0.768 |
| 30 days post-operative | 18 | 1.9 (7.9) | 7 | 4.8 (12.6) | -0.4 (-9.6 - 8.8) | 0.929 |
| 90 days post-operative | 17 | 7.8 (14.6) | 8 | 4.2 (11.8) | 7.1 (-4.0 - 18.2) | 0.209 |

*Larger values represent better functioning/QoL for domains D1 to D6. Smaller values represent less negative symptoms for domains (D7 to D15).

†Repeated measures analysis using a Generalised Estimating Equation regression model adjusted for baseline score, age and gender.

**Supplementary Table 3: QLQ-CR29 scores - Number (%) of patients with moderate to severe functioning problems or symptoms over time.**

|  | **Robotic** | | **Laparoscopic** | | **Robotic - Laparoscopic** |
| --- | --- | --- | --- | --- | --- |
| **Scale/Timepoint** | **N** | **n (%)** | **N** | **n (%)** | **Difference in %'s**  **(95% CI)** |
| Body image scale |  |  |  |  |  |
| Baseline | 18 | 1 (5.6) | 7 | 0 |  |
| Discharge | 15 | 1 (6.7) | 6 | 2 (33.3) | -26.7 (-69.5 - 22.5) |
| 30 days post operative | 18 | 1 (5.6) | 7 | 1 (14.3) | -8.7 (-49.8 - 34.1) |
| 90 days post operative | 17 | 1 (5.9) | 8 | 2 (25.0) | -19.1 (-57.3 - 23.1) |
| Anxiety scale |  |  |  |  |  |
| Baseline | 18 | 5 (27.8) | 7 | 1 (14.3) |  |
| Discharge | 15 | 6 (40.0) | 6 | 2 (33.3) | 6.7 (-41.1 - 52.8) |
| 30 days post operative | 18 | 4 (22.2) | 7 | 1 (14.3) | 7.9 (-35.2 - 49.1) |
| 90 days post operative | 17 | 3 (17.6) | 8 | 1 (12.5) | 5.1 (-34.4 - 45.0) |
| Weight scale |  |  |  |  |  |
| Baseline | 18 | 2 (11.1) | 7 | 1 (14.3) |  |
| Discharge | 15 | 2 (13.3) | 6 | 0 | 13.3 (-35.0 - 58.4) |
| 30 days post operative | 18 | 0 | 7 | 0 |  |
| 90 days post operative | 17 | 1 (5.9) | 8 | 2 (25.0) | -19.1 (-57.3 - 23.1) |
| Sexual interest |  |  |  |  |  |
| Baseline | 17 | 16 (94.1) | 7 | 6 (85.7) |  |
| Discharge | 14 | 13 (92.9) | 5 | 4 (80.0) | 12.9 (-37.4 - 62.1) |
| 30 days post operative | 17 | 16 (94.1) | 7 | 6 (85.7) | 8.4 (-34.6 - 50.3) |
| 90 days post operative | 15 | 14 (93.3) | 8 | 7 (87.5) | 5.8 (-35.9 - 47.4) |
| Urinary frequency scale |  |  |  |  |  |
| Baseline | 18 | 5 (27.8) | 7 | 4 (57.1) |  |
| Discharge | 15 | 3 (20.0) | 6 | 4 (66.7) | -46.7 (-81.9 - 2.3) |
| 30 days post operative | 18 | 4 (22.2) | 7 | 4 (57.1) | -34.9 (-71.6 - 9.6) |
| 90 days post operative | 17 | 5 (29.4) | 8 | 4 (50.0) | -20.6 (-59.8 - 23.1) |
| Blood and mucus in stool scale |  |  |  |  |  |
| Baseline | 18 | 1 (5.6) | 7 | 0 |  |
| Discharge | 15 | 0 | 6 | 1 (16.7) | -16.7 (-64.1 - 31.6) |
| 30 days post operative | 18 | 0 | 7 | 0 |  |
| 90 days post operative | 16 | 0 | 8 | 0 |  |
| Stool frequency scale |  |  |  |  |  |
| Baseline | 18 | 1 (5.6) | 6 | 0 |  |
| Discharge | 15 | 2 (13.3) | 6 | 1 (16.7) | -3.3 (-49.2 - 43.4) |
| 30 days post operative | 17 | 1 (5.9) | 7 | 1 (14.3) | -8.4 (-50.3 - 34.6) |
| 90 days post operative | 17 | 1 (5.9) | 8 | 0 | 5.9 (-34.3 - 45.0) |
| Urinary Incontinence scale |  |  |  |  |  |
| Baseline | 18 | 0 | 7 | 0 |  |
| Discharge | 15 | 0 | 6 | 0 |  |
| 30 days post operative | 18 | 0 | 7 | 0 |  |
| 90 days post operative | 17 | 1 (5.9) | 8 | 0 | 5.9 (-34.3 - 45.0) |
| Dysuria scale |  |  |  |  |  |
| Baseline | 18 | 0 | 7 | 0 |  |
| Discharge | 15 | 0 | 6 | 0 |  |
| 30 days post operative | 18 | 0 | 7 | 0 |  |
| 90 days post operative | 16 | 0 | 8 | 1 (12.5) | -12.5 (-54.0 - 32.1) |
| Abdominal Pain scale |  |  |  |  |  |
| Baseline | 18 | 3 (16.7) | 7 | 1 (14.3) |  |
| Discharge | 15 | 6 (40.0) | 6 | 2 (33.3) | 6.7 (-41.1 - 52.8) |
| 30 days post operative | 18 | 2 (11.1) | 7 | 0 | 11.1 (-31.4 - 51.5) |
| 90 days post operative | 17 | 1 (5.9) | 8 | 0 | 5.9 (-34.3 - 45.0) |
| Buttock Pain scale |  |  |  |  |  |
| Baseline | 18 | 0 | 7 | 0 |  |
| Discharge | 15 | 1 (6.7) | 6 | 0 | 6.7 (-41.1 - 52.8) |
| 30 days post operative | 18 | 1 (5.6) | 7 | 1 (14.3) | -8.7 (-49.8 - 34.1) |
| 90 days post operative | 17 | 0 | 8 | 0 |  |
| Bloating scale |  |  |  |  |  |
| Baseline | 18 | 4 (22.2) | 7 | 1 (14.3) |  |
| Discharge | 15 | 9 (60.0) | 6 | 3 (50.0) | 10.0 (-37.6 - 55.0) |
| 30 days post operative | 18 | 2 (11.1) | 7 | 1 (14.3) | -3.2 (-45.0 - 39.2) |
| 90 days post operative | 17 | 2 (11.8) | 8 | 1 (12.5) | -0.7 (-39.7 - 39.7) |
| Dry Mouth scale |  |  |  |  |  |
| Baseline | 18 | 2 (11.1) | 7 | 1 (14.3) |  |
| Discharge | 15 | 5 (33.3) | 6 | 2 (33.3) | 0.0 (-47.0 - 47.0) |
| 30 days post operative | 18 | 2 (11.1) | 7 | 2 (28.6) | -17.5 (-58.6 - 26.1) |
| 90 days post operative | 17 | 1 (5.9) | 8 | 2 (25.0) | -19.1 (-57.3 - 23.1) |
| Hair Loss scale |  |  |  |  |  |
| Baseline | 18 | 1 (5.6) | 7 | 0 |  |
| Discharge | 15 | 0 | 6 | 0 |  |
| 30 days post operative | 18 | 1 (5.6) | 7 | 0 | 5.6 (-36.6 - 46.6) |
| 90 days post operative | 16 | 2 (12.5) | 8 | 0 | 12.5 (-32.1 - 54.0) |
| Taste scale |  |  |  |  |  |
| Baseline | 18 | 0 | 7 | 0 |  |
| Discharge | 15 | 0 | 6 | 1 (16.7) | -16.7 (-64.1 - 31.6) |
| 30 days post operative | 18 | 0 | 7 | 0 |  |
| 90 days post operative | 16 | 0 | 8 | 1 (12.5) | -12.5 (-54.0 - 32.1) |
| Flatulence scale |  |  |  |  |  |
| Baseline | 18 | 1 (5.6) | 6 | 1 (16.7) |  |
| Discharge | 15 | 5 (33.3) | 6 | 0 | 33.3 (-16.0 - 77.7) |
| 30 days post operative | 17 | 2 (11.8) | 7 | 0 | 11.8 (-32.3 - 53.1) |
| 90 days post operative | 17 | 3 (17.6) | 8 | 1 (12.5) | 5.1 (-34.4 - 45.0) |
| Faecal Incontinence scale |  |  |  |  |  |
| Baseline | 18 | 0 | 6 | 0 |  |
| Discharge | 15 | 0 | 6 | 1 (16.7) | -16.7 (-64.1 - 31.6) |
| 30 days post operative | 17 | 0 | 7 | 0 |  |
| 90 days post operative | 17 | 0 | 8 | 0 |  |
| Sore Skin scale |  |  |  |  |  |
| Baseline | 18 | 0 | 6 | 0 |  |
| Discharge | 15 | 2 (13.3) | 6 | 0 | 13.3 (-35.0 - 58.4) |
| 30 days post operative | 17 | 2 (11.8) | 7 | 0 | 11.8 (-32.3 - 53.1) |
| 90 days post operative | 17 | 1 (5.9) | 8 | 0 | 5.9 (-34.3 - 45.0) |
| Embarrassment scale |  |  |  |  |  |
| Baseline | 18 | 0 | 6 | 0 |  |
| Discharge | 15 | 2 (13.3) | 6 | 2 (33.3) | -20.0 (-64.1 - 28.9) |
| 30 days post operative | 17 | 1 (5.9) | 7 | 1 (14.3) | -8.4 (-50.3 - 34.6) |
| 90 days post operative | 17 | 0 | 8 | 0 |  |
| Impotence scale |  |  |  |  |  |
| Baseline | 6 | 3 (50.0) | 4 | 0 |  |
| Discharge | 7 | 4 (57.1) | 4 | 0 | 57.1 (-9.0 - 93.2) |
| 30 days post operative | 6 | 3 (50.0) | 4 | 2 (50.0) | 0.0 (-60.5 - 60.5) |
| 90 days post operative | 5 | 1 (20.0) | 3 | 0 | 20.0 (-52.4 - 77.0) |
| Dyspareunia scale |  |  |  |  |  |
| Baseline | 6 | 0 | 3 | 0 |  |
| Discharge | 5 | 0 | 2 | 0 |  |
| 30 days post operative | 7 | 0 | 2 | 0 |  |
| 90 days post operative | 9 | 1 (11.1) | 3 | 0 | 11.1 (-56.5 - 71.7) |

**Supplementary Table 4: EQ-5D-5L scales over time – Number (%) of patients with moderate to severe problems.**

|  | **Robotic** | | **Laparoscopic** | | **Robotic - Laparoscopic** |
| --- | --- | --- | --- | --- | --- |
| **Dimension/Time-point** | **N** | **n (%)** | **N** | **n (%)** | **Difference in %'s**  **(95% CI)** |
| Your mobility today |  |  |  |  |  |
| Baseline | 18 | 2 (11.1) | 7 | 1 (14.3) |  |
| Discharge | 15 | 4 (26.7) | 6 | 2 (33.3) | -6.7 (-52.8 - 41.1) |
| 30 days post-operative | 18 | 2 (11.1) | 7 | 0 | 11.1 (-31.4 - 51.5) |
| 90 days post-operative | 18 | 2 (11.1) | 8 | 0 | 11.1 (-31.0 - 51.2) |
| Your self-care today |  |  |  |  |  |
| Baseline | 18 | 0 | 7 | 0 |  |
| Discharge | 15 | 2 (13.3) | 6 | 1 (16.7) | -3.3 (-49.2 - 43.4) |
| 30 days post-operative | 18 | 0 | 7 | 0 | 0 |
| 90 days post-operative | 18 | 0 | 8 | 0 | 0 |
| Your usual activities today |  |  |  |  |  |
| Baseline | 18 | 1 (5.6) | 7 | 0 |  |
| Discharge | 14 | 7 (50.0) | 6 | 3 (50.0) | 0 (-46.9 - 46.9) |
| 30 days post-operative | 18 | 3 (16.7) | 7 | 3 (42.9) | -26.2 (-65.7 - 18.3) |
| 90 days post-operative | 18 | 3 (16.7) | 8 | 0 | 16.7 (-25.7 - 55.9) |
| Your pain/discomfort today |  |  |  |  |  |
| Baseline | 18 | 3 (16.7) | 7 | 1 (14.3) |  |
| Discharge | 15 | 3 (20.0) | 6 | 4 (66.7) | -46.7 (-81.9 - 2.3) |
| 30 days post-operative | 18 | 2 (11.1) | 7 | 0 | 11.1 (-31.4 - 51.5) |
| 90 days post-operative | 18 | 2 (11.1) | 8 | 1 (12.5) | -1.4 (-41.6 - 40.3) |
| Your anxiety/depression today |  |  |  |  |  |
| Baseline | 18 | 4 (22.2) | 7 | 0 |  |
| Discharge | 15 | 1 (6.7) | 6 | 1 (16.7) | -10.0 (-55.0 - 37.6) |
| 30 days post-operative | 18 | 3 (16.7) | 7 | 1 (14.3) | 2.4 (-40.2 - 44.2) |
| 90 days post-operative | 18 | 1 (5.6) | 8 | 1 (12.5) | -6.9 (-46.7 - 35.3) |

**Supplementary Table 5: EQ-5D-5L VAS scores^*^ over time**

|  | **Robotic** | | **Laparoscopic** | | **Robotic - Laparoscopic** | |
| --- | --- | --- | --- | --- | --- | --- |
| **Time-point** | **N** | **Mean (SD)** | **N** | **Mean (SD)** | **Adjusted Difference**  **(95% CI)^†^** | **P^†^** |
| Overall Health Scale |  |  |  |  |  |  |
| Baseline | 18 | 73.3 (18.2) | 7 | 82.1 (16.5) |  |  |
| Discharge | 15 | 63.7 (19.7) | 6 | 56.7 (23.2) | 7.3 (-11.5 - 26.1) | 0.446 |
| 30 days post-operative | 18 | 77.4 (9.8) | 7 | 74.3 (19.9) | 5.5 (-7.8 - 18.7) | 0.420 |
| 90 days post-operative | 18 | 76.9 (15.6) | 8 | 79.0 (14.5) | -1.0 (-10.7 - 8.6) | 0.831 |

*For the VAS 100 means the best that you can imagine, 0 worst you can imagine.

†Repeated measures analysis using Generalised Estimating Equation regression model adjusted for baseline score, age and gender.

**Supplementary Table 6: Brief Pain Inventory^*^ over time**

|  | | **Robotic** | | **Laparoscopic** | | **Robotic - Laparoscopic** | |
| --- | --- | --- | --- | --- | --- | --- | --- |
|  | | **n** | **Mean (SD)** | **n** | **Mean (SD)** | **Difference in means**  **(95% CI)^†^** | **P^†^** |
| Pain severity score | Day 1 | 16 | 4.0 (2.0) | 7 | 4.2 (2.4) | -0.2 (-2.2 - 1.8) | 0.842 |
|  | Day 3 | 18 | 3.3 (2.3) | 7 | 3.9 (1.9) | -0.7 (-2.7 - 1.4) | 0.509 |
|  | Day 5 | 15 | 2.9 (1.9) | 7 | 2.7 (1.7) | 0.2 (-1.6 - 1.9) | 0.840 |
| Pain Interference score | Day 1 | 16 | 3.9 (2.4) | 7 | 5.2 (4.1) | -1.3 (-4.1 - 1.5) | 0.332 |
|  | Day 3 | 17 | 3.2 (2.6) | 7 | 4.7 (4.0) | -1.5 (-4.3 - 1.4) | 0.293 |
|  | Day 5 | 15 | 3.0 (2.7) | 7 | 3.3 (2.9) | -0.3 (-3.0 - 2.3) | 0.808 |

*Higher scores indicate greater severity and more interference.

†t-test

**Supplementary Table 7: NASA Load Index scores**

|  | **Robotic (N=18)** | | **Laparoscopic (N=9)** | | **Robotic - Laparoscopic** | |
| --- | --- | --- | --- | --- | --- | --- |
|  | **n** | **Mean (SD)** | **n** | **Mean (SD)** | **Difference in Means (95% CI)^*^** | **P^*^** |
| **Mental Demand:** |  |  |  |  |  |  |
| How mentally demanding was the task | 18 | 42.7 (24.6) | 9 | 46.7 (23.5) | -4.0 (-24.4 - 16.4) | 0.689 |
| Weight | 16 | 3.9 (1.0) | 7 | 3.4 (1.5) | 0.5 (-0.6 - 1.6) |  |
| Adjusted Score | 16 | 169.7 (126.6) | 7 | 165.0 (124.9) | 4.7 (-114.2 - 123.5) |  |
| **Physical Demand:** |  |  |  |  |  |  |
| How physically demanding was the task | 18 | 16.1 (12.5) | 9 | 43.9 (26.8) | -27.8 (-43.3 - -12.4) | 0.001 |
| Weight | 16 | 1.1 (1.0) | 7 | 2.3 (1.9) | -1.2 (-2.5 - 0.0) |  |
| Adjusted Score | 16 | 13.1 (13.7) | 7 | 132.9 (154.3) | -119.8 (-198.3 - -41.3) |  |
| **Temporal Demand:** |  |  |  |  |  |  |
| How hurried or rushed was the pace of the task? | 18 | 18.9 (17.4) | 9 | 21.1 (21.8) | -2.2 (-18.1 - 13.7) | 0.776 |
| Weight | 16 | 1.3 (1.0) | 7 | 0.9 (1.5) | 0.5 (-0.6 - 1.5) |  |
| Adjusted Score | 16 | 27.4 (41.9) | 7 | 24.3 (60.0) | 3.2 (-41.8 - 48.1) |  |
| **Performance:** |  |  |  |  |  |  |
| How successful were you in accomplishing what you were asked to do? | 18 | 23.1 (16.2) | 9 | 44.4 (32.4) | -21.3 (-40.4 - -2.3) | 0.030 |
| Weight | 16 | 4.2 (0.8) | 7 | 3.6 (1.4) | 0.6 (-0.3 - 1.5) |  |
| Adjusted Score | 16 | 96.4 (68.0) | 7 | 135.7 (144.8) | -39.3 (-130.2 - 51.5) |  |
| **Effort:** |  |  |  |  |  |  |
| How hard did you have to work to accomplish your level of performance? | 18 | 36.3 (20.3) | 9 | 48.9 (22.0) | -12.6 (-30.1 - 5.0) | 0.154 |
| Weight | 16 | 3.0 (1.2) | 7 | 3.0 (1.2) | 0.0 (-1.1 - 1.1) |  |
| Adjusted Score | 16 | 111.7 (86.9) | 7 | 151.4 (87.8) | -39.7 (-121.9 - 42.4) |  |
| **Frustration:** |  |  |  |  |  |  |
| How insecure discouraged irritated stressed and annoyed were you? | 18 | 25.8 (19.6) | 9 | 36.1 (26.8) | -10.3 (-29.0 - 8.3) | 0.264 |
| Weight | 16 | 1.5 (1.7) | 7 | 1.9 (1.3) | -0.4 (-1.9 - 1.1) |  |
| Adjusted Score | 16 | 48.4 (64.0) | 7 | 92.9 (87.4) | -44.4 (-111.8 - 22.9) |  |
| **Overall:** |  |  |  |  |  |  |
| Sum of adjusted ratings | 16 | 466.7 (283.1) | 7 | 702.1 (219.4) | -235.5 (-486.6 - 15.7) |  |
| Overall workload score - Sum of adjusted ratings divided by 15 | 16 | 31.1 (18.9) | 7 | 46.8 (14.6) | -15.7 (-32.4 - 1.0) | 0.065 |

*t-test
